# Recent developments in antiviral agents against enterovirus 71 infection

**DOI:** 10.1186/1423-0127-21-14

**Published:** 2014-02-12

**Authors:** Chee Wah Tan, Jeffrey Kam Fatt Lai, I-Ching Sam, Yoke Fun Chan

**Affiliations:** 1 Department of Medical Microbiology, Faculty of Medicine, University of Malaya, 50603 Kuala Lumpur, Malaysia; 2 Tropical Infectious Disease Research and Education Center (TIDREC), Faculty of Medicine, University of Malaya, 50603 Kuala Lumpur, Malaysia

**Keywords:** Enterovirus 71, Enterovirus, Hand, foot and mouth disease, Neurological complications, Antiviral, Virus replication cycle

## Abstract

Enterovirus 71 (EV-71) is the main etiological agent of hand, foot and mouth disease (HFMD). Recent EV-71 outbreaks in Asia-Pacific were not limited to mild HFMD, but were associated with severe neurological complications such as aseptic meningitis and brainstem encephalitis, which may lead to cardiopulmonary failure and death. The absence of licensed therapeutics for clinical use has intensified research into anti-EV-71 development. This review highlights the potential antiviral agents targeting EV-71 attachment, entry, uncoating, translation, polyprotein processing, virus-induced formation of membranous RNA replication complexes, and RNA-dependent RNA polymerase. The strategies for antiviral development include target-based synthetic compounds, anti-rhinovirus and poliovirus libraries screening, and natural compound libraries screening. Growing knowledge of the EV-71 life cycle will lead to successful development of antivirals. The continued effort to develop antiviral agents for treatment is crucial in the absence of a vaccine. The coupling of antivirals with an effective vaccine will accelerate eradication of the disease.

## Introduction

Human enterovirus A71 (EV-71) belongs to the Enterovirus genus within the family of *Picornaviridae*. The EV-71 genome is a single-stranded, positive sense RNA with approximately 7411 nucleotides, and consists of an open reading frame flanked by 5′ and 3′ untranslated regions (UTRs) [1]. Internal ribosome entry site (IRES)-dependent translation initiates synthesis of the viral polyprotein, which is subsequently cleaved into structural proteins (VP1-VP4) and non-structural proteins (2A-2C and 3A-3D). The RNA genome is enclosed in an icosahedral capsid assembled from 60 copies of each of the four structural proteins [2].

EV-71 was first described in 1969, after its isolation from a two-month-old infant with aseptic meningitis in California, USA. Several EV-71 epidemics with high mortality rates occurred in Bulgaria and Hungary in 1975 and 1978 [3-5], respectively. Since then, many EV-71 outbreaks have been reported in Taiwan [6], Australia [7], Singapore [8], Malaysia [9], China [10-14], Vietnam [15] and Cambodia [16].

EV-71 infections usually manifest as mild hand, foot and mouth disease (HFMD), characterized by fever, mouth ulcers, and vesicles on the palms and feet. Unlike other HFMD-related enteroviruses, EV-71 also causes severe neurological manifestations, such as poliomyelitis-like acute flaccid paralysis and brainstem encephalitis in infants and children below 6 years old [17,18]. The fatal brainstem encephalitis is characterized by rapid progression of cardiopulmonary failure. Patients with neurological involvement who survive often have permanent neurological sequelae, with delayed neurodevelopment and reduced cognitive function [19,20].

Similar to the global poliovirus (PV) eradication initiative, an EV-71 vaccine is likely to be the most effective way to control, and hopefully eradicate disease [21,22]. Several promising EV-71 vaccine candidates are currently under clinical trial [23]. Nevertheless, effective antivirals are still needed for treatment of infected patients with severe disease [21,22]. This review will highlight the potential targets for EV-71 antivirals as well as recent developments and future prospects of antivirals against EV-71 infections.

## Review

### EV-71 virus life cycle

Similar to other viruses, EV-71 infection begins with initial attachment to attachment factors present on the cell surface, followed by interaction with entry receptors. EV-71 enters the cells through clathrin-mediated endocytosis and uncoats in the early endosomes. The viral RNA undergoes IRES-dependent translation, and the polyprotein is cleaved by 2A and 3C proteases into structural and non-structural proteins. Non-structural proteins are mainly involved in negative-sense and positive-sense RNA synthesis. The positive-sense viral RNA is then packed into the procapsid, which finally matures into infectious viral particles. Details of the EV-71 replication steps will be discussed according to their therapeutic targets [18,21,22].

### Therapeutics targeting viral attachment and entry

Virus-host receptor interaction is the first essential event during virus infection. The ability to recognize and bind to specific receptors determines the host range and tissue tropism [24]. Cell surface carbohydrates such as heparan sulfate glycosaminoglycan and sialic acid are often targeted by pathogens as attachment factors. EV-71 uses cell surface heparan sulfate [25] and sialylated glycan [26,27] as attachment receptors, which could concentrate the virus on the host cell surface and therefore enhance infectivity. Further interaction with entry receptors is required to initiate infection. Two functionally important entry receptors have been identified, scavenger receptor class B2 (SCARB2) and P-selectin glycoprotein ligand-1 (PSGL-1) [28,29]. SCARB2 is expressed in all cell types and regarded as the major EV-71 entry receptor. At low endosomal pH, SCARB2 is needed to induce viral uncoating [30,31]. Human SCARB2 transgenic mice infected with EV-71 showed lethal neurological manifestations with pathological features similar to humans and monkeys, suggesting that SCARB2 contributes to its pathogenesis [32,33]. PSGL-1 is only present on neutrophils and leukocytes. EV-71 binds to PSGL-1 and enters the cells through the caveolar endocytosis pathway [34]. Transgenic mice expressing human PSGL-1 failed to enhance EV-71 infectivity, suggested that PSGL-1 alone does not contribute to its pathogenesis [35].

Since host-receptor interactions are the first event during infection, inhibitors that block this event could act as potential therapeutics. The soluble form of cellular receptors could act as molecular decoys of cell-associated receptors. Soluble SCARB2, PSGL-1, sialic acid and heparin or heparin mimetics have been demonstrated to exhibit inhibitory effects against EV-71 infection *in vitro*[25,26,28,30,36]. Highly sulfated suramin and its analog, NF449, exhibited antiviral activity against EV-71 infection [25,37]. NF449-resistant mutants consist of two mutations in VP1, E98Q and K244R, implying that NF449 inhibited EV-71 infection by binding to the VP1 protein [37]. Similarly, kappa carrageenan, a sulfated polysaccharide from seaweed, also exhibited significant antiviral activity through targeting EV-71 attachment and entry [38]. The mechanism of these soluble decoys is possibly by disruption of the integrity of the EV-71 capsid structure or steric hindrance of receptor interactions.

Receptor antagonists could also be developed as potential antiviral agents. A peptide derived from EV-71 VP1, designated SP40 peptide (Ac-QMRRKVELFTYMRFD-NH_2_), was found to exhibit significant antiviral activity against different strains of EV-71 by blocking viral attachment to the cell surface heparan sulfate [39]. An anti-heparan sulfate peptide (Ac-MPRRRRIRRRQK-NH_2_), previously identified by Tiwari *et al.*[40], also inhibited EV-71 infection [25]. Another antimicrobial peptide, lactoferrin, also exhibited anti-EV-71 properties *in vitro* and *in vivo* through blocking viral attachment to the cell surface [41-43].

### Therapeutics targeting viral uncoating

The proposed EV-71 uncoating event involves attachment to the entry receptor, triggering a series of conformational changes resulting in A-particle formation that is primed for genome release. A second uncoating event occurs after endocytosis, and an unknown trigger causes RNA expulsion from the A-particles via the 2-fold axis, leaving behind an empty capsid [44]. Formation of the 135S A-particle happens in the presence of SCARB2 receptors and a low pH environment, suggesting that the A-particle is formed in the early endosomes [30,31]. Uncoating inhibitors (pocket binders) have been intensively studied as antiviral agents against many picornaviruses, including rhinovirus [45], PV [45], echovirus [46] and coxsackievirus [47]. The complex of WIN51711 with the EV-71 hydrophobic pocket underneath the canyon depression has recently been resolved by X-ray crystallography [48]. The key success factor of these uncoating inhibitors is their ability to fit into the VP1 hydrophobic pocket, stabilize the capsid structure, and therefore block the receptor-induced uncoating mechanism [48].

A series of modified WIN compounds including BPROZ-194, BPROZ-112, BPROZ-284, BPROZ-103, BPROZ-299, BPROZ-101, BPROZ-033, and BPROZ-074 were effective against EV-71 infection with IC_50_ values ranging from 0.8 nM to 1550 nM [49-54]. However, a single point mutation in VP1 V192M was sufficient to confer resistance to BPROZ-194 [51]. Other than modified WIN compounds, the broad spectrum enterovirus inhibitor pleconaril also inhibited EV-71 infection *in vitro* and *in vivo*[55,56]. However, pleconaril failed to inhibit the cytopathic effect induced by a Taiwan 1998 EV-71 isolate [49]. Another group of capsid binders, pyridazinyl oxime ethers chemically derived from pirodavir such as BTA39 and BTA188, significantly inhibited EV-71 infection [57]. Crystallographic studies showed the pirodavir predecessor R61837 complexed with rhinovirus 14 by binding to the hydrophobic pocket underneath the canyon floor, similar to the mechanism of WIN compounds [58]. 4′,6-Dichloroflavan (BW683C), previously identified as an anti-rhinovirus compound, was also effective against EV-71 infection [59,60]. Mechanistic studies demonstrated that BW683C binds to and stabilizes rhinovirus to heat or acid inactivation, implying that BW682C acts as viral uncoating inhibitor [61-63].

### Therapeutics targeting viral RNA translation

EV-71 protein synthesis commences with translation initiation of the cap-independent IRES element at the 5′UTR of the EV-71 genome [64]. IRES is a *cis-*acting element that forms tertiary RNA structures and requires assistance from IRES-specific *trans*-acting factors (ITAFs) to recruit other cellular translation machinery to the viral RNA. The EV-71 open reading frame (ORF) is translated into a single polyprotein, which is subsequently processed by virus-encoded proteases 2A and 3C into the structural capsid proteins (VP1-VP4) and the nonstructural proteins (2A-2C and 3A-3D) mainly involved in the replication of the viral RNA [65].

The antisense-mediated mechanism consists of oligonucleotides (8-50 nucleotides in length) that bind to RNA through Watson-Crick base pairing and modulate the function of the targeted RNA [66]. RNA interference (RNAi) involves the cleavage of targeted mRNA through the RNA-induced silencing complex. Small interfering RNA (siRNA) targeting highly conserved regions of 5′UTR [67], VP1, VP2 [68], 2C, 3C, 3D [69,70], and 3′UTR [69] significantly inhibited EV-71 infection in a dose-dependent manner. In addition, short hairpin RNA (shRNA) was effective against EV-71 infection *in vitro* and *in vivo*[70-72]. The use of siRNA in clinical settings is hampered by its short half-life in plasma. Improved siRNA with 2′O methylation and 2′ fluoro modifications have recently been demonstrated against EV-71 infection [67]. However, siRNA also has poor endosomal uptake which limits the clinical application of these siRNAs. Other translation suppressing nucleotides, for example, peptide conjugated phosphodiamidate morpholino oligomers (PPMO) showed promising results in inhibiting PV and coxsackievirus B3 [73,74]. Unlike siRNA or shRNA, PPMO interacts with targeted RNA, especially the IRES region, and blocks ribosome recruitment and therefore inhibits viral RNA translation [66]. PPMO readily penetrates the cells and is resistant to nuclease degradation. Our unpublished data confirms that PMO are highly effective against EV-71.

Compounds that down-regulate the activity of IRES-dependent translation could potentially be developed into antiviral agents. Quinacrine, which impairs IRES-dependent translation by preventing the interaction between polypyrimidine-tract binding protein (PTB) and IRES, has been demonstrated to act against EV-71 infection [75]. Kaempferol, a flavonoid, was found to inhibit EV-71 IRES activity by altering the composition of ITAFs [76]. Geniposide derived from *Fructus gardeniae* inhibited EV-71 replication via inhibition of viral IRES activity [77]. Amantadine, a tricyclic symmetric amine previously used against influenza A virus infection, was found to suppress EV-71 IRES translation [78-80].

### Therapeutics targeting viral polyprotein processing

Maturation cleavage of polyprotein into different viral proteins is a critical step during EV-71 infection. EV-71 2A and 3C protease are the key proteases that cleave the viral precursor polyprotein into each of the component proteins required for viral replication and packaging. Interestingly, EV-71 2A and 3C proteases suppress type I interferon by targeting mitochondrial anti-viral signaling (MAVS) protein and melanoma differentiation associated gene (MDA-5) viral recognition receptor signaling [81,82]. Since EV-71 2A and 3C proteases are involved in multiple roles in EV-71 infection and evasion of host innate immunity, they are important potential targets for development of antiviral therapeutics.

A pseudosubstrate, LVLQTM peptide, could inhibit EV-71 infection through binding to the active site of 2A protease [83]. Rupintrivir (AG7088) is an irreversible peptidomimetic inhibitor of human rhinovirus 3C protease, which reached phase 2 clinical trials with promising outcomes [84-89]. Rupintrivir showed significant inhibition of EV-71 infection *in vitro* and *in vivo* but with reduced efficacy as compared with human rhinoviruses [90-93]. X-ray crystallography of the complex of EV-71 3C protease with rupintrivir revealed that the half-closed S2 sub-site and the size reduced S1′ pocket of EV-71 3C protease limits the access of the rupintrivir’s P1′ group which contains a lactam ring [94,95]. A series of 3C protease rupintrivir analogues were designed based on AG7088, with an aldehyde replacement of the α,β-unsaturated ester. Compound 10b significantly inhibited EV-71 infection [96]. An orally bioavailable 3C protease inhibitor, designated as compound 1, also exhibited antiviral activities against multiple rhinovirus serotypes and enteroviruses *in vitro*[89]. Flavonoids such as fisetin and rutin, have also been identified as 3C protease inhibitors [97].

### Therapeutics targeting the membranous viral RNA replication complex and other host factors

The genomic replication of enteroviruses has been shown to occur in membranous compartments in the cytoplasm. The membranous vesicles induced during PV infection have been reported to be associated with autophagy signalling [98,99]. These compartments resemble the autophagosomes and consist of viral proteins as well as microtubule-associated protein 1 light chain 3-II (LC3-II). LC3-II is the membrane-bound form of LC3 that serves as the marker of autophagy induction [100]. During PV infection, these double-membrane vesicles consist of viral particles that undergo autophagic maturation typically characterized by LC3-II co-localization with lysosomal-associated membrane protein 1 (LAMP1) [100]. Similarly, EV-71 induces autophagy formation in RD and SK-N-SH cells, and association between autophagosome-like vesicles and EV-71 VP1 in neurons of the cervical spinal cords of mice was observed [101]. The authors concluded that autophagic signalling induced by EV-71 is crucial for EV-71 replication. This provides an alternative antiviral strategy for EV-71 to target host factors related to autophagy that are crucial for viral replication.

The discovery of antiviral drugs is mainly based on virus targets. The high replication and mutation rates of enteroviruses may generate resistance to these direct-acting antivirals. Targeting host factors may establish a higher genetic barrier to resistance and can be used in combination with viral inhibitors. The compound GW5074, a Raf-1 inhibitor, has been shown to influence EV-71 viral yield [37,102]. Activation of the Raf-1/ERK pathway in host cells induces autophagy signalling [103]. The downstream transducer of this pathway, BNIP3 competes with Beclin 1 for binding with Bcl-2 during autophagy induction [104]. GW5074 may impair autophagy activation through the inhibition of the Raf-1/ERK pathway. Thus, the replication of EV-71 that requires autophagosome formation may be inhibited in the presence of the GW5074 compound. Heat shock protein 90 beta (HSP90β), an isomer of HSP90, has been reported to have crucial roles in EV-71 entry and assembly. Geldanamycin (GA) and its analog, 17-allyamino-17-demethoxygeldanamycin (17-AAG), inhibit HSP90β activities and protect hSCARB2 transgenic mice from the challenge with EV-71 [105].

Inhibitors that target host factors such as those involved in cellular autophagy and HSP90β could be used against multiple EV-71 genotypes and enterovirus serotypes, due to their similar pathways of replication [106,107]. The major drawbacks of these inhibitors that target host factors are specificity and cellular toxicity. Therefore, there is an unmet need to develop specific and non-toxic antivirals that impair the cellular autophagy pathway and HSP90β during EV-71 infection.

The amino acid sequences of the non-structural proteins of EV-71 are highly conserved and have more than 60% similarity to PV. Two hydrophobic regions are found in the 2B viral protein of PV and are pivotal for its viroporin functionality [108]. 2B viroporin mediates the integration of viral protein into the ER membrane and this increases the membrane permeability to promote virus release [108]. A study has reported that EV-71 2B protein might mediate a chloride-dependent current in oocytes. A chloride-dependent current inhibitor, 4,4′-diisothiocyano-2,2′-stilbenedisulfonic acid (DIDS) has been reported to inhibit EV-71 infection in RD cells [109]. The 2C viral protein of PV consists of Walker A, B and C motifs that are homologous to the motifs found in NTP-binding proteins or in members of the helicase superfamily III [110]. An amphipathic helix domain is located at the N-terminal of 2C viral protein that has the function of promoting oligomerization [110]. Recently, two antiviral compounds, metrifudil (N-(2-methylphenyl) methyl adenosine) and N^6^-benzyladenosine, blocked EV-71 replication via interaction with 2C viral protein or 2BC precursor protein [37]. Mutants resistant to metrifudil had a mutation in the 2C viral protein (E325G), while N^6^-benzyladenosine-resistant mutants had double mutations at the 2C viral protein (H118Y and I324M) [37]. However, the mechanism of inhibition is yet to be determined. Both MRL-1237 and TBZE-029, derivatives of benzimidazole, exhibit antiviral activity against various enteroviruses, and have been identified to target the picornaviral 2C viral protein [111,112]. Both of these derivatives may exert potent antiviral activity against EV-71 since EV-71 and PV shared high similarity in all the non-structural proteins. Guanidine hydrochloride is an extensively-studied picornavirus inhibitor [113,114], which inhibits the replication of PV [115,116], coxsackieviruses [117], echoviruses, and foot-and-mouth disease virus [118]. Interestingly, guanidine hydrochloride also inhibits EV-71 infection and a single mutation, M193L at the 2C protein was sufficient to confer resistance [119]. This agent is likely to prevent the association of 2C/2BC with host membrane structures during viral replication [120].

The 3A viral protein of PV contains hydrophobic domains that facilitate its binding with membranous vesicles induced during viral RNA replication [121,122]. A benzimidazole derivative, enviroxime exhibits potent activity against PV and rhinovirus by interacting with 3A viral protein [119]. Strong antiviral effects of enviroxime have been shown against EV-71 [123]. Bifunctional inhibitors AN-12-H5 and AN-23-F6, are enviroxime-like compound that also targets 3A, VP1 and VP3, inhibits EV-71 infection efficiently [124]. However, the precise mechanism of action by enviroxime and AN-12-H5 against EV-71 infection remains unknown. Another compound, TTP-8307, was identified as a potent 3A inhibitor that significantly inhibited CV-A16 infection, with reduced activity against EV-71 [112].

### Therapeutics targeting RNA-dependent RNA polymerase (RdRP) complex

The viral RNA replication of enteroviruses begins with the linkage of genomic RNA with the 3B protein (VPg) at the 5′ end to form the uridylylated state of VPg (VPg-pUpU). Additionally, VPg uridylylation is stimulated by the viral precursor protein 3CD [125]. The positive strand of viral RNA is used as a template to synthesize the negative strand, which in turn serves as the template for the synthesis of new positive strands. The synthesis of both positive and negative strands of viral RNA is primed by VPg-pUpU [126]. Nucleotide site 311 of the RNA-dependent RNA polymerase (RdRP) of EV-71 is pivotal for VPg uridylylation and viral RNA synthesis, as mutations here impair the binding of VPg to RdRP, but did not influence normal RdRP activity [127].

Ribavirin (1-β-D-ribofuranosyl-1,2,4-triazole-3-carboxyamine) is a conventional nucleoside analogue that targets the RdRP of picornaviruses [128]. Ribavirin inhibits EV-71 infection with an IC_50_ of 266 μM, and prevents EV-71 induced paralysis and death in mice [129]. Recently, a piperazine-containing pyrazolo [3,4-*d*] pyrimidine derivative, DTriP-22, was shown to effectively target the RdRP of EV-71 with IC_50_ values of 0.15 – 0.98 μM, and suppress the accumulation of both positive and negative strands of viral RNA during EV-71 infection. DTriP-22-resistant mutants had mutations in the RdRP, implying that DTriP-22 interacts with RdRP and inhibits poly (U) elongation activity, but not VPg uridylylation [130].

## Conclusion

**Figure 1:**
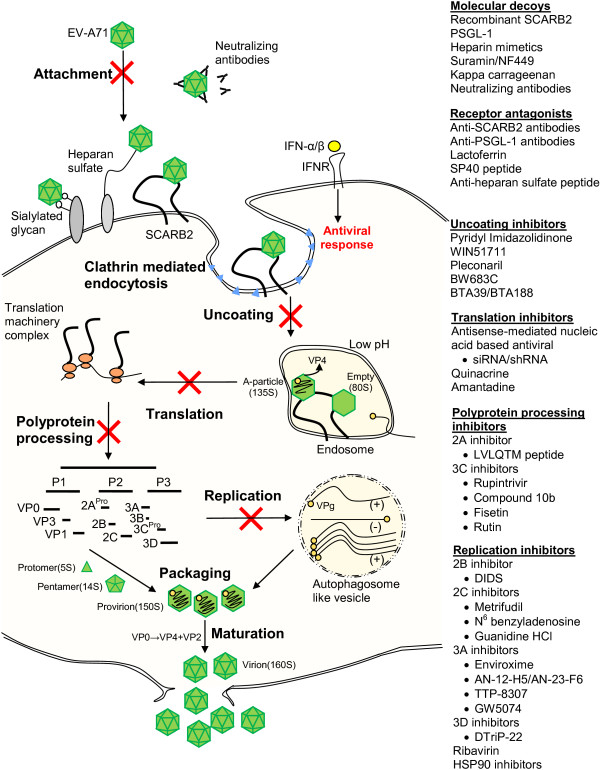
**Schematic illustration of EV-71 intracellular infection and summary of the antiviral agents.** The antiviral agents are classified according to their mechanism of actions, which include molecular decoys, receptor antagonists, uncoating inhibitors, translation inhibitors, polyprotein processing inhibitors and replication inhibitors.

Figure 1 and Table 1 summarizes all the potential targets of antivirals and lists the recent antiviral agents with significant antiviral activities against EV-71 infection as discussed above. Amongst these drugs, modified WIN compounds are antivirals with the lowest IC_50_. Only bovine lactoferrin, pleconaril, shRNA, siRNA, rupintrivir, ribavirin and 17-AAG have been tested *in vivo.* Ribavirin and amantadine are already in clinical use for other viruses, and rupintrivir and pleconaril are in clinical development.

**Table 1:** **List of antivirals against EV-71 infection tested *in vitro *** **and *in vivo***

**Antivirals**	**EV-71 genotype tested**	**IC**_**50**_**/EC**_**50**_	***In vitro *** **cell type**	**Resistant mutants**	***In vivo *** **mouse model**	**Reference**
Therapeutics targeting viral attachment and entry						
Molecular decoys						
Recombinant SCARB2	B3	N/R	RD			[28]
PSGL-1	C2	N/R	L-PSGL-1.1			[29]
Heparin mimetics						
Heparin	C2	205 μg/ml	Vero, RD			[25,36]
Heparan sulfate	C2	290 μg/ml	Vero			[36]
Pentosan polysulfate	C2	238 μg/ml	Vero			[36]
Dextran sulfate	B4	N/R	RD			[25]
Suramin/NF449	B1, B3, B4	6.7 μM	RD	VP1 E98Q, K244R		[25,37]
Kappa carrageenan	B4	N/R	Vero			[38]
Enviroxime-like compounds						
AN-12-H5	B1	0.55 μM	RD	VP1 M119L, VP3 R227K		[124]
AN-23-F6	B1	0.15 μM	RD	VP1 A224T		[124]
Receptor antagonists						
Anti-SCARB2 antibodies	B3	N/R	RD			[28]
Anti-PSGL-1 antibodies	B3, B4, C1, C2, C4	N/R	Jurkat			[29]
Bovine lactoferrin	C2, MP4^a^	10.5 – 24.5 μg/ml	RD, Vero, SK-N-SH		17-days old ICR	[42,43]
Human lactoferrin	N/R	103.3 – 185.0 μg/ml	RD, Vero			[42]
SP40 peptide	A, B4, C2	6 – 9.3 μM	RD, HeLa, HT-29, Vero			[39]
Anti-heparan sulfate peptide	B4	N/R	RD			[25,40]
Therapeutics targeting viral uncoating						
Pyridyl imidazolidinone						
BPROZ-299	C2	0.02 μM	RD	VP1 V192M		[52]
BPROZ-284	A, B1, C2	0.04 μM	RD			[49]
BPROZ-194	C2	1.552 μM	RD	VP1 V192M		[51,52]
BPROZ-160	C2	0.011 μM	RD	VP1 V192M		[52]
BPROZ-112	A, B1, C2	0.04 μM	RD			[49]
BPROZ-103	C2	0.13 μM	RD	VP1 V192M		[52]
BPROZ-101	A, B1, C2	0.0012 μM	RD			[52,53]
BPROZ-074	A, B1, C2	0.0008 – 0.018 μM	RD	VP1 V192M		[52,54]
BPROZ-033	A, B1, C2	0.0088 – 0.069 μM	RD			[52,54]
WIN51711	B3	N/R	RD			[48]
Pleconaril	A	0.13-0.54 μg/ml	RD		1-day old ICR	[56]
BW683C	A	> 10 μM	HEp-2			[59]
Compound 3 g	A	0.45 μM	HEp-2			[59]
BTA39	A	0.001 μM	Vero			[57]
BTA188	A	0.082 μM	Vero			[57]
Therapeutics targeting viral translation						
RNA-based therapeutics						
siRNA	B4	< 1 nM	RD		1-day old Balb/c	[67-72]
shRNA	B4	< 1 nM	RD		1-day old Balb/c	[67-72]
Quinacrine	C4	9.71 μM	RD			[75]
Amantadine	Pseudo-EV-71	N/R	COS-1			[78]
Therapeutics targeting viral polyprotein processing						
2A inhibitor						
LVLQTM peptide	C4	9.6 μM	HeLa			[83]
3C inhibitors						
Rupintrivir	C4	0.014 μM	RD		2-days old ICR	[93]
Compound 10b	C2	0.018 μM	RD			[96]
Fisetin	CMUH01*	85 μM	RD			[97]
Rutin	CMUH01*	110 μM	RD			[97]
Therapeutics targeting viral replication						
2B inhibitor						
DIDS	C4	N/R	RD			[109]
2C inhibitors						
Metrifudil	B1	1.3 μM	RD	2C E325G		[37]
N^6^ benzyladenosine	B1	0.1 μM	RD	2C H118Y, I324M		[37]
Guanidine-HCl	B3	N/R	RD	2C M193L		[119]
3A inhibitors						
Enviroxime	A	0.15 μM	Vero			[112]
AN-12-H5	B1	0.55 μM	RD	3A E39G		[124]
AN-23-F6	B1	0.15 μM	RD			[124]
TTP-8307	A	> 60 μM	Vero			[112]
GW5074	B1	6.4 μM	RD			[124]
3D inhibitors						
DTriP-22	A, B2, C2	0.3 μM	RD	3D R163K		[130]
Ribavirin	C2, M2^b^	266 μM	RD, SK-N-SH, N18	3D G64R, S264L	12-days old ICR	[129]
Heat-shock protein 90 inhibitor						
Geldanamycin	B4, C2	N/R	RD			[105]
17-AAG	C2, C4	N/R	N/R		7-days old hSCARB-Tg C57BL/6 mice	[105]

^a^Mouse-adapted EV-71 strain Tainan/4643/98 (C2); ^b^Mouse-adapted EV-71 strain derived from MP4 with additional two passages in mice; *EV-71 strain with unidentified genotype; and N/R means not reported.

RD: rhabdomyosarcoma cells; Vero: African green monkey kidney cells; SK-N-SH: human neuroblastoma cells; N18: mouse neuroblastoma cells; HeLa: human cervical adenocarcinoma epithelial cells; HT-29: human colon adenocarcinoma cells; HEp-2: HeLa contaminant cells; Jurkat: human T lymphocytes cells; and COS-1: monkey kidney fibroblast cells.

The availability of a suitable animal model carrying all the required receptors and attachment factors for testing of the antivirals will accelerate the development of antivirals. The clinical use of other antiviral agents has been hampered by the potential adverse effects to the host and emergence of drug resistance mutants. Combination therapy targeting different replication steps of EV-71 infection cycle has shown synergistic activity [131] and could minimize the emergence of antiviral resistance. A new antiviral strategy to screen all licensed drugs against EV-71 infection would be more promising for clinical use. Other newer antivirals that act as immunomodulators and lethal mutagens offer a new strategy for development of antivirals. With the endemic and epidemic nature of EV-71, the continued efforts to develop antiviral agents for prophylaxis or treatment are crucial in the absence of a vaccine. Together with an effective vaccine, eradication of EV-71 is anticipated.

## Abbreviations

EV-71: Enterovirus 71; HFMD: Hand, foot and mouth disease; IRES: Internal ribosome entry site; ITAF: IRES-specific *trans*-acting factor; MAVS: Mitochondrial anti-viral signaling; MDA-5: Melanoma differentiation associated gene; ORF: Open reading frame; PV: Poliovirus; RdRP: RNA-dependent RNA polymerase; VPg: Viral protein genome-linked.

## Competing interests

CWT and YFC have a pending patent on SP40 peptide.

## Authors’ contributions

CWT, JKFL, ICS and YFC drafted the manuscript. All authors read and approved the final manuscript.
